# Assessment of the Strength Parameters of the Quadriceps Femoris Muscles in Polish University Students after a 3-Week Program of Neuromuscular Electrical Stimulation Using the RSQ1 Method

**DOI:** 10.3390/ijerph182111717

**Published:** 2021-11-08

**Authors:** Aleksandra Rywacka, Małgorzata Stefańska, Alicja Dziuba-Słonina

**Affiliations:** 1 Department of Physiotherapy in Neurology and Pediatrics, Wroclaw University of Health and Sport Sciences, 51-612 Wroclaw, Poland; alicja.dziuba-slonina@awf.wroc.pl; 2 Department of Physiotherapy in Dysfunctions of the Locomotor System, Wroclaw University of Health and Sport Sciences, 51-612 Wroclaw, Poland; malgorzata.stefanska@awf.wroc.pl

**Keywords:** electrical muscle stimulation, neuromuscular electrical stimulation, RSQ1 method, exercise program

## Abstract

A rehabilitative program for patients who lose strength and muscle mass along with the ability to perform intensive exercises is lacking. We developed a 3-week training program based on neuromuscular electrical stimulation (NMES) using a RSQ1 device (modulated current resulting from the overlapping of two-component currents) for RSQ1 electrostimulation to improve strength parameters of the quadricep femoris muscles and compare its effectiveness to isometric training. Nineteen university students were randomly divided into the NMES group (10 sessions) and the control group who trained. We measured the circumference of the thigh, as well as peak torques of the flexor and extensor muscles before and after the start and after the end of the training program. Both tested training programs gave similar results. Differences between measured parameters were not significant except for differences in the peak torques of the knee flexors (9.9% for left limb; *p* = 0.2135 vs. 7.8% for rift limb; *p* = 0.2135) and the circumference of the left thigh—2% for both (left *p* = 0.5839 and right *p* = 0.1088). Comparable results of the tested training programs suggest that NMES is a good alternative for people who cannot perform exercises, but want to maintain or improve their physical fitness.

## 1. Introduction

In the 21st century, technological progress in medicine and physiotherapy, as well as the holistic approach to patients and athletes, have created new opportunities for rehabilitation and training [1,2]. Electrical muscle stimulation (EMS) treatments, in addition to their wide application in rehabilitation, are more and more often used in training sessions to diversify strength exercises or as an alternative for people who want to maintain physical fitness but do not have time or opportunities for standard training in the gym [3,4,5,6,7]. Kemmler et al. [7], in their randomised controlled study, showed that neuromuscular electrical stimulation (NMES) sessions correspond to four times longer training with high-intensity exercises. After the use of NMES, jumping ability is improved [8] and maximum muscle strength is increased [9]. A series of properly selected EMS sessions leads to an enlargement of the muscle’s cross-sectional area, a significant increase in its maximum strength, and also accelerated muscle activation [10,11]. Research confirms that EMS causes regeneration of skeletal muscles by reducing the level of oxidative stress in the muscle’s satellite cells and by forming new myofibrils [12]. NMES methods, which stimulate muscles innervated by the motor nerves, are currently very popular, with the RSQ1 device (Physicare International B.V., Zaandam, The Netherlands) being one of the most innovative methods. The novelty of this method (named after the device, an RSQ1 method) is rooted in several features. The RSQ1 method is an NMES, in which the muscles innervated by the motor nerves are stimulated. In contrast, transcutaneous electrical nerve stimulation (TENS) stimulates the sensory nerves that are responsible for transmitting pain signals to the brain. During the TENS procedure pain signals are inhibited by stimulating the sensory nerves, while during RSQ1 treatments, the muscle contracts engaging a larger number of muscle fibres during training or regeneration after exercise.

Kots and Chilon conducted studies concerning the use of NMES for healthy people as early as 1970, and their publication appeared in 1975 [13]. However, to date, despite such rapidly developing technology, no model of conduct has been developed that would transparently and in an available manner provide a use for this training tool [14]. Although there are many more interesting and widely available EMS and NMES methods on the market, their effectiveness in healthy people has not yet been sufficiently studied and analysed. This is because, currently, there are many different EMS methods available with different parameters, which cause different reactions in subjects regarding the applied stimulation and causes different results of these tests [6]. One of the researchers who focused on a specific type of EMS (WB-EMS) is Kemmler et al. [7,15]. Given the potential benefits of NMES, together with the lack of evidence on its effectiveness, we attempted to evaluate this tool in a training program. The aim of this study was to develop a 3-week training program based on NMES using RSQ1 electrostimulation to improve strength parameters of the quadricep femoris muscles in university students, and to compare its effectiveness to classical strength training. We hypothesised that the RSQ1 method can give similar results to standard training in a gym, so we intended to investigate the results in this comparative study.

## 2. Materials and Methods

### 2.1. Subjects

Overall, 19 university students from the University School of Physical Education, Wroclaw, Poland (11 women and 8 men) participated in the study. Before qualifying for the study, each participant had to meet inclusion or exclusion criteria. In addition, each participant had to complete a questionnaire containing information such as age, sex, body weight, body height, whether there had been an injury in the last 3 years in the area of the lower limbs and how many hours a week the participant spends on physical activity. The following inclusion criteria were considered: age from 22 to 30 years, active lifestyle (maximum of 3–4 h of training per week), no injuries in the area of the lower limbs in the last 3 years, no chronic diseases, and informed consent to participate in the study. The criteria for exclusion from the study included: a current injury in the lower limbs or an injury that occurred more than 3 years from the start of the experiment, competitive or professional performing of sports, and also other contraindications for performing EMS, i.e., psychogenic symptoms, pregnancy, neurological deficits, cardiovascular diseases, respiratory diseases, pacemakers, and cancer. The subjects were informed about the course of the research and gave their written informed consent to participate in the study. Participants were randomly divided into two groups. The study group consisted of 10 people (7 women and 3 men), who underwent a 3-week training program (10 sessions) using the RSQ1 method. The control group consisted of 9 students (4 women and 5 men), who performed a training program at home consisting of 10 sessions. Detailed characteristics of both groups are presented in Table 1. The project was approved by the Senate Ethics Committee of University School of Physical Education in Wroclaw and was conducted in accordance with the Declaration of the World Medical Association. Research and therapy using the RSQ1 method were carried out in the research laboratory at the Faculty of Physiotherapy at the University School of Physical Education in Wroclaw.

### 2.2. Study Procedure

RSQ1 therapy lasted 3 weeks and consisted of 10 sessions. In the first week, each subject participated in 4 treatment sessions, and in the second and third weeks—3 sessions. Each participant, during the given exercises, was subjected to EMS of the quadricep femoris muscles in the right and left limbs simultaneously for 30 min.

**Figure 1:**
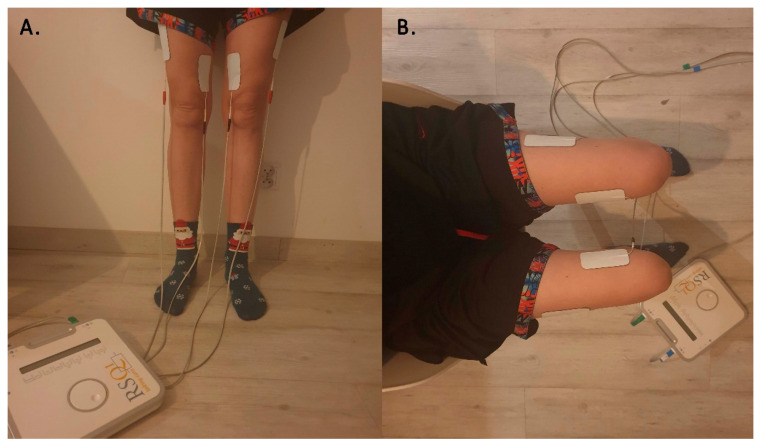
Placement of electrodes of the thigh and the RSQ1 device: (**A**) front view and (**B**) from above view.

The RSQ1 device has two electrical circuits, each ending in two electrodes (size 5 × 9 cm): an anode and a cathode. The electrodes were made using the MultiStick Gel technology, thanks to which they are durable and can be used repeatedly. The patented MultiStick^®^ Gel technology uses an adhesive multi-layer gel and eliminates problems with the performance caused by using one layer of gel. It ensures increased flexibility and better comfort by adapting to the surface of the skin while maintaining excellent current-conducting properties. On the outside, it is covered with a non-woven fabric that facilitates application and gives full comfort for long-term use. The procedure was always performed symmetrically, and two electrodes were glued to each lower limb (in the middle of the quadricep femoris muscles)—the negative electrode (cathode) on the vastus medialis muscle, and the positive electrode (anode) on the vastus lateralis muscle. The placement of the electrodes was constant for the duration of the program. The placement of the electrodes is shown in Figure 1.

As the current flowed through the RSQ1 device, the participants performed a simple and repetitive exercise. The participant used both legs during the exercises. From the first to the fourth therapy, there were dynamic exercises: standing up and sitting down using a chair—SIT/STAND (StS). During the exercises, the feet were placed parallel at a distance of 25–30 cm from each other, and the knee joints were bent from 0 to 90 degrees. The movement was performed symmetrically and axially. The exercise was repeated 5 times in the first two sessions, and 10 times in the next two sessions.

From the 5th to the 10th therapies, apart from dynamic exercises, the students also performed static (isometric) exercises—WALL SQUAT and EXTREME HAM GLUT (WS/ExHGl). The static work of the muscles was generated during the squat against the wall—the so-called “chair”. The participants held this position in the 5th and 6th sessions for 30 s, in the 7th and 8th sessions for 40 s, and in the 9th and 10th sessions for 50 s. They were then standing up and bending their torso forward with their legs straightened at the knees and their quadriceps muscles tensed. They held this position for 1 min. Each exercise was repeated 5 times by the participants.

The control group performed a training program (10 sessions) for 3 weeks at home. Exercises during the first 4 sessions (first week) were the same as in the SIT/STAND (StS) sessions, but without EMS. The task of the participants was to slowly perform the exercise 20 times. From the 5th to the 10th sessions (2nd and 3rd week), the participants in the control group performed WALL SQUAT and EXTREME HAM GLUTE (WS/ExHGl). The time of this exercise did not change—it was always 60 s in the “chair” position, and 60 s bending forward. The exercises were carefully selected to avoid too much stimulus in the form of intense exercise [16].

### 2.3. Study Device

The RSQ1 device (Physicare International B.V., Zaandam, The Netherlands) is a CE IIa class medical device. It can be operated only by doctors, physiotherapists, or therapeutic massage technicians after training in the principles of RSQ1 application and obtaining a certificate. An RSQ1 device is equipped with an intelligent system that controls the maximum available voltage that can be used for stimulation. The RSQ1 device was developed in the Netherlands for high-performance athletes for training and therapeutic purposes. RSQ1 differs from other EMS therapies with regards to three aspects. RSQ1 treatments can be used as therapeutic therapies, but also as training sessions. During RSQ1 treatment, two types of currents overlap: a low-frequency current of 40–500 Hz, and a medium-frequency current of 1000–10,000 Hz. Due to this, their properties are combined. During the RSQ1 session, electrodes are placed on both limbs, and the exercises are symmetrical [17]. During the flow of currents, the participant performed exercises that were individually selected according to his/her needs [15,17]. RSQ1 protocols depend on the place where the procedure is performed and the group of people (whether they are healthy or injured). For this reason, we included the knee joint and healthy people in the described studies.

Indications for the RSQ1 treatment include the need for the increase of muscle mass and moment of strength, restoration of function and the appropriate range of mobility in the joints, promotion of blood circulation, reduction in swelling, prevention of thrombosis. Additionally, the need for an alternative to strength training can be considered. People with a pacemaker, heart diseases (arrhythmia, infarction), neoplastic diseases, deep vein thrombosis, and pregnant women are contraindicated for this treatment. There have been no reported side effects.

### 2.4. Study Measures

Before the starting and after ending the training program, measurements of the circumference of the thigh were performed in both study groups with the use of anthropometric tape. The measurement, which was performed in a standing position, was made 10 and 20 centimetres above the medial edge of the knee joint (U10 and U20). In order to increase the reliability of the result, the measurement was performed by the same person, and the result itself was the average of three measurements [18]. The assessment of the peak torque of the flexor and extensor muscles in both knee joints were assessed twice for each participant using the BiodexSystem3 measuring system (Biodex Medical Systems, Inc., New York, NY, USA). The static operating mode of the device was used. The axis of the measuring device was set so that it coincided with the axis of the rotation of the joint—the angle in the joint was set at 75°. The test was performed using a measurement protocol consisting of two series, in which the static work time and the break time lasted for five seconds.

The analysed parameters were:

Peak Torque (PKTQ [Nm])—peak (maximum) torque,

AGON/ANTAG RATIO—agonist to antagonist momentum ratio.

### 2.5. Statistical Analysis

The analysis was performed using Statistica 13.3 software (StatSoft, Tulsa, OK, USA). The Shapiro–Wilk test was used to check the distribution of all the analysed parameters, which in most cases turned out to be close to the normal distribution. Descriptive statistics were calculated—average, median and standard deviation. The non-parametric Wilcoxon pairwise test was used to compare the results obtained before and after the therapy, and the Mann-Whitney U test was used to compare the results obtained from the study group and the control group. A confidence level of 95% was adopted. In order to check whether both applied treatment methods are equivalent, the power value was calculated for the proportions calculated for the results obtained before and after the therapy in both study groups. The Calculator Power and Sample Size to Compare 2 Proportions: 2-Sample Equivalence (http://powerandsamplesize.com/ (accessed on 10 February 2021)) was used. δ value of 0.05 was considered as non-inferiority or superiority margin.

## 3. Results

The results of the tests carried out in the study group before and after treatment were the values of the peak torque measured in static conditions for the flexor muscles and extensor muscles of both knee joints. The values of the torque ratio of the antagonistic flexor and extensor muscles were calculated, and the circumferences of the thigh (10 and 20 cm above the base of the patella) were measured.

When analysing the results of the study group (RSQ1), higher values of the peak torque, torque ratio, as well as a slight enlargement of the circumferences were observed after the therapy. The differences recorded for the flexor muscles of the joint, and the circumferences measured for the left limb turned out to be statistically significant (Table 2).

In the control group, the average results of all the analysed parameters turned out to be higher in study 2, but no statistically significant differences were found (Table 3).

When comparing the results obtained in both groups, both before and after therapy, no statistically significant differences were found in the strength parameters and circumferences (Table 4).

## 4. Discussion

In the present study, we aimed evaluate our 3-week training program with RSQ1 electrostimulation to improve strength parameters of the quadriceps femoris muscles and to compare its effectiveness to classic strength training. TheRSQ1 method consisted of simple repetitive exercises that were performed together with simultaneous EMS sessions. In the control group, an exercise program, but without stimulation, was used. Despite different methods in both groups, similar final results were observed in most of the measured parameters. The exceptions were the statistically significant differences in the peak torques of the knee flexors, and also in the circumference of the left thigh in the study group. Our results are in line with other reports from the literature which showed that EMS increased strength parameters [6,9,15]. The RSQ1 method is a novel tool with little research [19] but it has the potential to be used as an alternative that can help to improve physical fitness. This study has to be considered preliminary but the promising results justify future research.

A similar tendency was noticed by Kemmler et al. in 2016 [7], who compared the effect of a 16-week whole body (WB)-EMS program with high-intensity (resistance) training on the body composition and strength of healthy men. Researchers observed a comparable, or at least similar effect, of 16-week training programs on the strength of the knee extensors and torso extensors. However, the authors emphasise that there was a significant difference between the groups in the total duration of treatments (WB-EMS 403 ± 37 min versus high-intensity training 847 ± 87 min), which shows that the WB-EMS training resulted in a comparable improvement in the tested parameters with more than two times shorter training time. In the present study, an EMS program of the RSQ1 method was used, and the obtained results were comparable to the results of the control group, which was subjected to training with an optimal load so that both men and women could perform exercise. The RSQ1 method can therefore be a good alternative for a wide group of people after surgery or injuries, as well as for obese or elderly patients who cannot participate in intensive strength training. Such people often cannot perform intense exercises due to postoperative oedema, a limited range of motion, or intense pain. RSQ1 therapy enables appropriate strength and muscle mass to be maintained or reconstructed without overloading the joints.

The aim of the study conducted by Dziuba-Słonina et al. [19] was to evaluate the effect of NMES using the ARP wave method combined with a high-protein diet on the thigh circumference of patients after a knee joint injury. ARP wave therapy consisted of 10 procedures, during which dynamic exercises (concentric and eccentric) were performed during the stimulation. As a result of the therapy, in a group of women and men, a more significant increase in the circumference of the limb after the injury was noticed when compared to the healthy limb. The present study included solely healthy people. When analysing thigh circumferences after the therapy, a statistically significant increase was only observed in the left (non-dominant) limb. This may prove the greater impact of the applied stimulus on weaker muscle groups, which in consequence leads to an increase in the symmetry of the circumferences. This was observed in both the present study and in the Dziuba-Słonina et al. study [19].

A similar relationship was observed when analysing the peak torque of the knee flexors and extensors. The strength abilities of the weaker group (flexors) after RSQ1 therapy increased significantly, thus improving the strength balance of the antagonist muscles. Maffiuletti et al. [9] presented the results of their research that was conducted on 12 well-trained Italian tennis players subjected to a 3-week quadriceps muscle electrostimulation program. It consisted of 9 sessions of 16 min before their tennis training. They observed the largest changes in maximal voluntary contraction values 3 weeks after the end of the experiment, and in the counter movement jump 2 weeks after the end of the study. In the present study, significant changes were only recorded in the values of the peak torque of the knee joint flexors after the training program was completed. This is perhaps related to the too short process of observing the participants, or to the use of a program of the RSQ1 method.

Wirtz et al. [20] conducted an experiment to demonstrate the effects of EMS on muscle strength, power, sprinting and jumping. The authors confirmed a statistically significant increase in strength, squat jump, counter movement jump and pendulum sprint performance in the group subjected to training and EMS, as well as in the control group subjected to training only (but without statistically significant differences between the groups). The authors conducted the assessment twice: before the training process and immediately after the last session, which may be the reason, as in the present study, for the lack of differences between the study group and the control group.

In 2019, Pano-Rodriguez et al. [21] carried out a systematic review of the literature on the impact of the application of EMS as a form of training in healthy people with a sedentary lifestyle. The purpose of this review was to identify the evidence on the effects of EMS treatments stimulating muscles of the upper and lower limbs simultaneously. Ultimately, 21 articles were included and analysed in which the circuits, strength parameters and blood parameters were examined. Most of the studies were carried out on small groups and changes in the measured circuits and force parameters were not statistically significant. The reason for the lack of significant changes could be the simultaneous EMS of too many muscle groups or taking a second measurement immediately after the end of the experiment, too low frequency of treatments per week or too short sessions. The results of the review suggest that more studies including people without injuries are needed to determine the effects of the various parameters used in EMS treatments. As the literature review shows, most studies look at people with injuries. Therefore, in the present study, we used the RSQ1 method locally in healthy people without previous injuries. Furthermore, we evaluated physically active people, but not professional athletes to demonstrate the effect of RSQ1 electrostimulation on the selected muscles of the lower limbs. The circumference of the thigh at two anthropometric points (C10, C20) and the strength parameters of the extensor and flexor muscles of the knee were analysed. The values of the analysed parameters did not show statistical significance. It may be due to too prompt the second measurement (immediately after the end of the experiment) or too few experimental groups. For this reason, in the future, we plan to continue the research on larger groups and perform the second measurement one week after the end of the experiment so that the muscles have time to regenerate. Safety and the lack of negative effects observed during WB-EMS therapy are very important issues [7,22]. This, in combination with the proven effect of stimulation on the increase in muscle mass in untrained people, regardless of their age, sex and muscle condition [19], with the reduction of back pain; with the reduction of the connective and abdominal adipose tissue (which is a risk factor for insulin resistance, atherosclerosis, hypertension) [23], and also with the improvement of dynamic and static balance [9] may suggest that EMS should be applied as one of the most frequently used elements of the therapeutic process in various dysfunctions.

In this study, several limitations should be taken into consideration. We examined a small but precisely selected group of people. All participants were healthy, young, and physically active. We did not evaluate different age groups, people with strength and endurance deficits, as well as with comorbidities. Nevertheless, such an approach resulted in the inclusion of a homogeneous study group.

In light of the promising results, the RSQ1 method can be considered useful for athletes who wish to diversify their training or as an alternative for elderly or obese people, where the disease causes a loss of strength and muscle mass, and at the same time, limits the ability to perform exercises with a load and a large number of repetitions. We plan to continue the research on the use of RSQ1. Future research will include a larger group of participants and more experimental groups, including a control group. We also plan to take into account the strength parameters. In a future project, however, we will focus not only on the strength parameters of the extensor and flexor muscles of the knee joint, but also on speed parameters and how the percentage ratio of flexor to extensor muscles changes.

## 5. Conclusions

Comparable results of the tested training programs suggest that NMES using the RSQ1 method is a good alternative for people who for various reasons cannot perform exercises with a load, but want to maintain or improve their physical fitness. The research should be continued on a larger group of participants with a bigger number of sessions or with a 6-week program and extended to measure speed parameters in dynamic work.

## Figures and Tables

**Table 1:** Characteristics of the tested groups.

	Study Group			Control Group			
	Average	SD	Median	Average	SD	Median	*p*
Age [years]	25.4	2.87	24	23.89	2.33	24	0.5136
Weight [kg]	79.5	9.55	81.5	78.33	7.12	80	0.9025
Height [cm]	177.8	5.09	178	181	4.67	180	0.3477

SD—standard deviation; *p*—coefficient of the Mann–Whitney U test.

**Table 2:** Descriptive statistics and the statistical significance of the differences registered between the analysed parameters before and after therapy in the study group treated using the RSQ1 device.

		Before Therapy			After Therapy			
		Average	Median	SD	Average	Median	SD	*p*
PT E [Nm]	R	224.69	233.70	54.01	242.41	227.10	58.89	0.2845
	L	227.66	222.00	60.77	235.99	223.40	52.21	0.3329
PT F [Nm]	R	97.45	98.55	21.97	105.74	97.70	28.00	0.0367 *
	L	91.95	90.25	24.75	102.12	101.35	29.62	0.0469 *
A/A1 [%]	R	42.02	40.60	7.60	44.22	45.05	9.78	0.2026
	L	42.90	44.70	8.35	43.63	43.75	10.06	0.5147
CIRCUIT 10 [cm]	R	51.05	51.00	3.37	51.65	52.25	3.61	0.1282
	L	51.10	51.50	3.25	52.05	53.00	3.32	0.0357 *
CIRCUIT 20 [cm]	R	59.20	60.50	4.62	60.05	60.75	4.25	0.0929
	L	59.50	60.50	3.62	60.40	61.00	4.08	0.0423 *

A/A1—PT F to PT E ratio; CIRCUIT 10—circumference of the thigh 10 cm above the base of the patella; CIRCUIT 20—circumference of the thigh 20 cm above the base of the patella; PT E—peak torque measured in static conditions for the extensor muscles; PT F—peak torque measured in static conditions for the flexor muscles; SD—standard deviation. * statistically significant value *p* < 0.05.

**Table 3:** Descriptive statistics and the statistical significance of the differences registered between the analysed parameters before and after therapy in the control group.

		Before Therapy			After Therapy			
		Average	Median	SD	Average	Median	SD	*p*
PT E [Nm]	R	230.62	222.20	71.64	231.52	237.70	65.58	0.9528
	L	247.90	249.50	53.51	251.33	236.00	46.21	0.5147
PT F [Nm]	R	107.07	104.00	18.30	111.63	110.30	18.40	0.2135
	L	105.83	96.00	25.80	113.46	108.90	18.62	0.2135
A/A1 [%]	R	48.72	48.00	10.15	50.79	46.80	12.50	0.8590
	L	42.73	42.20	4.38	45.51	43.90	5.13	0.0858
CIRCUIT 10 [cm]	R	50.56	51.00	3.13	50.83	51.00	3.04	0.1088
	L	50.17	51.00	3.34	50.28	50.00	3.36	0.5839
CIRCUIT 20 [cm]	R	58.11	60.00	3.79	58.11	60.00	3.69	1.0000
	L	57.94	59.00	3.40	57.83	59.00	3.61	0.5839

A/A1—PT F to PT E ratio; CIRCUIT 10—circumference of the thigh 10 cm above the base of the patella; CIRCUIT 20—circumference of the thigh 20 cm above the base of the patella; PT E—peak torque measured in static conditions for the extensor muscles; PT F—peak torque measured in static conditions for the flexor muscles; SD—standard deviation.

**Table 4:** Comparison of the results recorded in the study and control groups before and after the therapy.

Study Group v.s. Control Group	Before Therapy *p* Value		After Therapy *p* Value		1 − β	
	Right Limb	Left Limb	Right Limb	Left Limb	Right Limb	Left Limb
PT E [Nm]	0.9025	0.4379	0.8383	0.2703	−0.87	−0.79
PT F [Nm]	0.4877	0.4379	0.5956	0.2703	−0.86	−0.86
A/A1 [%]	0.1416	0.8065	0.4142	0.6831	−0.78	−0.83
CIRCUIT 10 [cm]	0.7751	0.5676	0.6831	0.2207	−0.42	−0.50
CIRCUIT 20 [cm]	0.4877	0.3272	0.3074	0.1914	−0.64	−0.67

A/A1—PT F to PT E ratio; CIRCUIT 10—circumference of the thigh 10 cm above the base of the patella; CIRCUIT 20—circumference of the thigh 20 cm above the base of the patella; PT E—peak torque measured in static conditions for the extensor muscles; PT F—peak torque measured in static conditions for the flexor muscles; 1 − β—power of the test.

## Data Availability

The data presented in this study are available within the article.
